# Heterogeneous Photocatalytic Degradation of Selected Pharmaceuticals and Personal Care Products (PPCPs) Using Tungsten Doped TiO_2_: Effect of the Tungsten Precursors and Solvents

**DOI:** 10.3390/molecules29174164

**Published:** 2024-09-03

**Authors:** Kunyang Li, Jing Li, Fengying Luo, Yuhua Yu, Yepeng Yang, Yizhou Li

**Affiliations:** Yunnan Key Laboratory of Metal-Organic Molecular Materials and Device, School of Chemistry and Chemical Engineering, Kunming University, Kunming 650214, China; 18340365616@163.com (K.L.); 15125354629@163.com (J.L.); 18187857956@163.com (F.L.); 18213659741@163.com (Y.Y.); yepengyang@kmu.edu.cn (Y.Y.)

**Keywords:** PPCPs, W^5+^/W^6+^ ratio, tungsten precursors, photodegradation performance, solvents

## Abstract

Pharmaceuticals and personal care products (PPCPs) which include antibiotics such as tetracycline (TC) and ciprofloxacin (CIP), etc., have attracted increasing attention worldwide due to their potential threat to the aquatic environment and human health. In this work, a facile sol-gel method was developed to prepare tungsten-doped TiO_2_ with tunable W^5+^/W^6+^ ratio for the removal of PPCPs. The influence of solvents in the synthesis of the three different tungsten precursors doped TiO_2_ is also taken into account. WCl_6_, ammonium metatungstate (AMT), and Na_2_WO_4_●2H_2_O not only acted as the tungsten precursors but also controlled the tungsten ratio. The photocatalyst prepared by WCl_6_ as the tungsten precursor and ethanol as the solvent showed the highest photodegradation performance for ciprofloxacin (CIP) and tetracycline (TC), and the photodegradation performance for tetracycline (TC) was 2.3, 2.8, and 7.8 times that of AMT, Na_2_WO_4_●2H_2_O as the tungsten precursors and pristine TiO_2_, respectively. These results were attributed to the influence of the tungsten precursors and solvents on the W^5+^/W^6+^ ratio, sample crystallinity and surface properties. This study provides an effective method for the design of tungsten-doped TiO_2_ with tunable W^5+^/W^6+^ ratio, which has a profound impact on future studies in the field of photocatalytic degradation of PPCPs using an environmentally friendly approach.

## 1. Introduction

Pharmaceuticals and personal care products (PPCPs), as a comprehensive category of emerging pollutants, have received increasing attention in recent years due to their diverse potential impacts on the dynamics of the natural environment and human health [1,2]. Generally, PPCPs include a broad range of medicinal and consumer chemicals such as antibiotics, non-steroidal anti-inflammatory drugs (NSAIDs), blood lipid regulators (BLRs), and fragrances. Tetracycline (TC) and ciprofloxacin (CIP) as a broad-spectrum antibiotic is widely used as a medicine in treatments [3]. Residues of TC and CIP enter the aquatic environment due to incomplete absorption and degradation. It is imperative to efficiently degrade the TC and CIP by employing advanced technology [4,5,6].

For the environmentally friendly treatment of TC and CIP, some sustainable methods such as adsorption [3], coagulation [4], biological treatment [5], and filtration [6] have attracted attention in recent years, but have been found to be ineffective. TiO_2_-based materials have been identified as promising candidates for the photocatalytic degradation of PPCPs in aquatic environments [7]. Nevertheless, the large bandgap energy (3.2 eV) and the accompanying suppression limit its practical applicability for natural solar applications [8,9,10,11]. In view of this, the doping of elements such as P [12], S [13], N [14], Fe [15], Cr [16], and Co [17] into pristine TiO_2_ has been investigated for the photocatalytic degradation of TC and CIP under natural solar light [18,19].

Transition metals have attracted extensive attention in the field of photocatalysis due to their unique photophysical-chemical properties [20]. Currently, tungsten (W) is widely used in photocatalysis [21], sensing [22], water decomposition [23], and photoelectrochemical properties [24]. Among these applications, it has shown high activity and environmental friendliness in photocatalytic degradation. TiO_2_ has also been doped with tungsten in order to enhance photocatalytic which optoelectrical properties are achieved by doping with different oxidation states (W^4+^, W^5+^ and W^6+^) [25]. Compared to W^6+^ doping, W^4+^ and W^5+^ doping has been studied less frequently. However, it has been proved that composites prepared with the participation of tungsten or tungsten compounds formed by W^5+^ and W^4+^ doping can be used to adjust the expected photocatalytic activity of tungsten-doped TiO_2_ [26,27]. W^5+^ and W^4+^ doped tungsten compounds can easily act as electron acceptors, thus improving the efficiency of photogenerated carrier separation [26,27].

The tungsten precursors are also essential for the production of tungsten-doped TiO_2_. In general, organic and inorganic tungsten compounds, such as sodium tungstate dehydrate (Na_2_WO_4_●2H_2_O), ammonium metatungstate (AMT), tungsten hexachloride (WCl_6_), and tungsten (VI) hexa-ethoxide (W(OC_2_H_5_)_6_ [28,29,30], have been widely used as tungsten precursors for the preparation of tungsten-doped TiO_2_. Different tungsten precursors have significant effects on the performance and properties of the prepared tungsten-doped TiO_2_ composite photocatalysts. Different tungsten precursors affect the distribution, particle size, and surface properties of tungsten during the reaction process, which in turn affect the activity of the photocatalysts [19].

It can be concluded that W^5+^ and W^6+^ co-doped TiO_2_ prepared by varying the tungsten dopants could further improve the photocatalytic activity of tungsten-doped TiO_2_. For example, Sanjayan Sathasivam et al. have demonstrated that the inclusion of tungsten in TiO_2_ materials in low quantities can enhance the photocatalytic activity by reducing the carrier mobility [25]. Raul Quesada-Cabrera et al. prepared W^5+^/W^6+^ coexistence WO_3_/TiO_2_ heterojunction films that exhibited unusual electron transfer from WO_3_ to TiO_2_ [31]. Tungstate-doped TiO_2_-SiO_2_ aerogels were prepared by the sol-gel method which contained W^5+^, W^6+^, and preferentially photodegraded methamphetamine [32]. Nevertheless, up to now there is little information available on the synergistic effects of the W^5+^/W^6+^ co-doping of TiO_2_, let alone the tuning of the W^5+^/W^6+^ ratios.

The solvent is also an important factor in the preparation of tungsten-doped TiO_2_. In general, considering factors such as solubility and hygroscopicity, methanol and ethanol are the most commonly used solvents employed in synthesis due to their water miscibility and compatibility with other compounds [33]. Less attention has been paid in the literature to the use of lower alcohols, especially in the sol-gel method for the synthesis of TiO_2_-based materials [34]. However, attention should be paid to the potential significance of the molecular configuration of the solvent in the sol-gel method. Previous studies also shown that the crystallinity, surface morphology, and optical properties of materials are strongly dependent on the solvent [34]. Zainab Yousif Shnain et al. have shown that the solvent has a significant effect on the particle size and morphology of the synthesized nanoparticles [35]. In the sol-gel process, the optical properties of the material are significantly affected by varying the solvent ratio of water to ethanol [36]. Pu-Xian Gao et al. selected six organic compounds as solvents for the synthesis of TiO_2_ by _S_olvotherma. The study implied that the configuration of the organic solvents can have a significant impact on the microstructures and properties of the final products [37]. Obviously, the complex nature of solvents, such as polarity and hydrogen bonding, can affect the kinetics of the sol-gel reaction and the properties of the material. There is a lack of reports regarding the effect of solvents during the synthesis of TiO_2_-based materials using the sol-gel method.

In this study, tungsten-doped TiO_2_ photocatalysts with varying W^5+^ and W^6+^ ratios were prepared via the sol-gel method at a low synthesis temperature. In the dark and photoreaction stages, the effects of different tungsten precursors and solvents on the adsorption and photocatalytic activity of TC and CIP were investigated. These results demonstrated that the photocatalytic activity of all W^5+^/W^6+^ co-doped TiO_2_ is superior to that of pristine TiO_2_. Furthermore, the photocatalytic activity of W^5+^/W^6+^ co-doped TiO_2_ under simulated sunlight increased with increasing W^5+^/W^6+^ by varying the tungsten precursors. Besides, the tungsten-doped TiO_2_ prepared with ethanol as a solvent showed enhanced photocatalytic activity compared to the samples prepared with DMF. We hypothesized that this could be attributed to the hydroxyl groups of the solvent molecules and the elemental electronegativity.

## 2. Results and Discussion

### 2.1. Characterization

**Figure 1:**
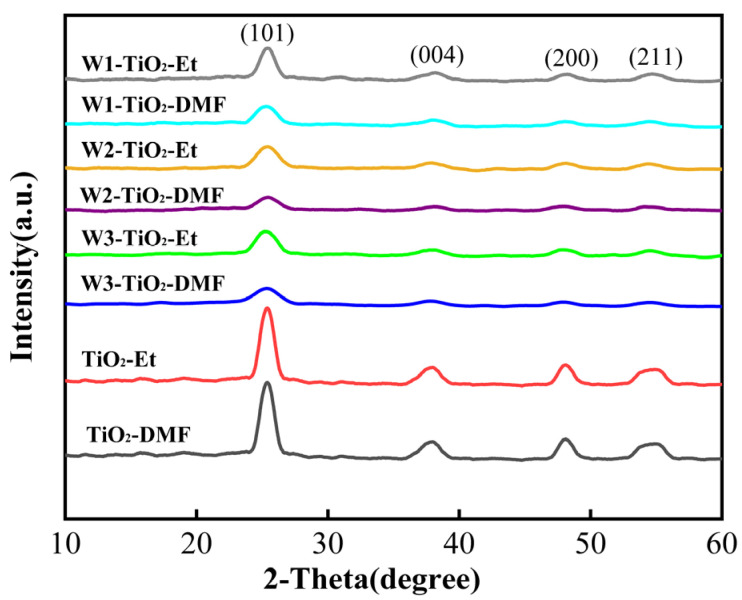
XRD patterns of pristine TiO_2_ and tungsten-doped TiO_2_.

XRD was used to analyse the crystalline phases of the prepared samples. As shown in Figure 1, the diffraction angles of the anatase phases corresponding to (101), (004), (200), and (211) of anatase crystals are 25.3°, 37.9°, 48.1°, and 55.1°, respectively, which proves that anatase structures are present in all TiO_2_-based materials. The diffraction peaks of the samples with dimethylformamide (DMF) as the solvent (diffraction peaks at 2 θ = 25.3°) are broader and weaker than those of the samples with ethanol (Et) as the solvent, which may indicate that the samples dissolved in ethanol have a better crystallisation effect compared to DMF. In addition, as shown in Table 1, we calculated the crystallinity of the samples and the results also indicate that the samples dissolved in ethanol have higher crystallinity. This revealed a preference for ethanol as a solvent in the crystallization of tungsten-doped TiO_2_ powders, over DMF. It is well known that crystallinity is crucial for photocatalytic activity. Materials with elevated crystallinity generally exhibit improved properties in photocatalysis attributed to the well-ordered and uniform structures, which facilitate charge transfer from the center to the surface [38]. The analysis indicates that tungsten-doped TiO_2_ synthesized utilizing WCl_6_ as the tungsten precursor potentially exhibits enhanced photocatalytic performance, attributed to its superior crystallinity, compared with Na_2_WO_4_ and AMT. For all samples, no phases other than the anatase phase were detected. The formation of WO_3_ was not evidenced by the characteristic peaks typically observed in XRD spectra, as noted by other authors. This may be due to the low concentration of WO_3_, which was insufficient for detection by XRD [29,39]. The absence of a characteristic WO_3_ peak in the XRD pattern of tungsten-doped TiO_2_ suggests that tungsten ions either formed W–O–Ti bonds within the lattice or occupied interstitial sites [40]. Several studies have shown that tungsten ions successfully substitute titanium ions within the TiO_2_ crystal lattice due to the similar ionic radius (W^6+^ at 0.060 nm and Ti^4+^ at 0.0605 nm) [40].

The grain size of the prepared material was calculated using the Scherrer formula. The results were presented in Table 1. As illustrated in Table 1, the average grain size of the tungsten-doped TiO_2_ samples is smaller than that of the un-doped TiO_2_ samples, indicating that doping with various tungsten precursors affected the crystallinity of materials and thus hinders the crystal growth.

**Figure 2:**
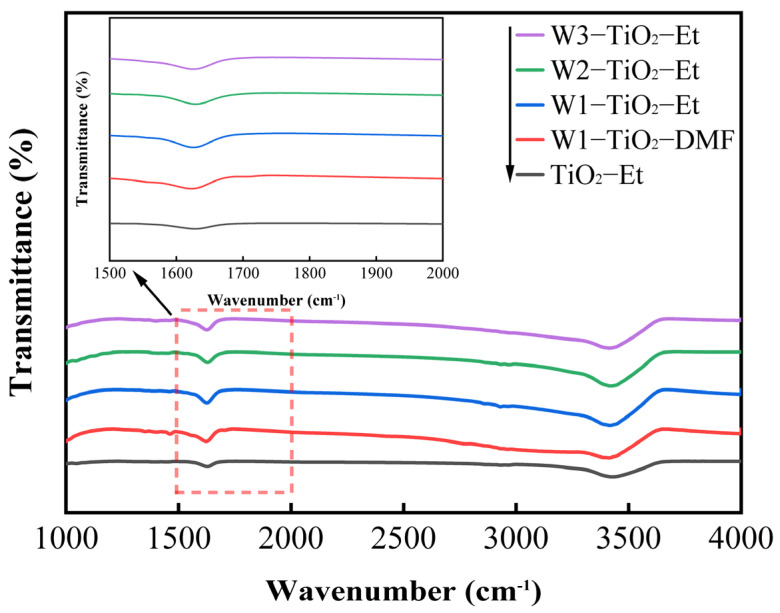
FTIR spectra of pristine TiO_2_ and tungsten-doped TiO_2_.

The FTIR spectra of the pristine TiO_2_ and the tungsten-doped TiO_2_ materials between 4000 cm^−1^ and 1000 cm^−1^ are shown in Figure 2. There are only two distinct absorption bands around 3450 cm^−1^ and 1630 cm^−1^ in Figure 2, which represent the stretching vibrations of the water and hydroxyl groups, respectively [41]. As illustrated in Figure 2, the introduction of tungsten results in the slight enhancement of the peaks at 3450 cm^−1^ and 1630 cm^−1^, suggesting an increase in the presence of water and hydroxyl groups, respectively. This observation implies that the addition of tungsten may lead to a higher concentration of hydroxyl groups within the tungsten-doped TiO_2_. The Lewis surface acidity of tungsten-doped TiO_2_ increases with the addition of tungsten [42], making it easier to adsorb water and form surface hydroxyl groups, while the –OH groups can capture photogenerated holes (h^+^) and convert them into active •OH radicals [43], thus improving the photocatalytic performance of the material.

**Figure 3:**
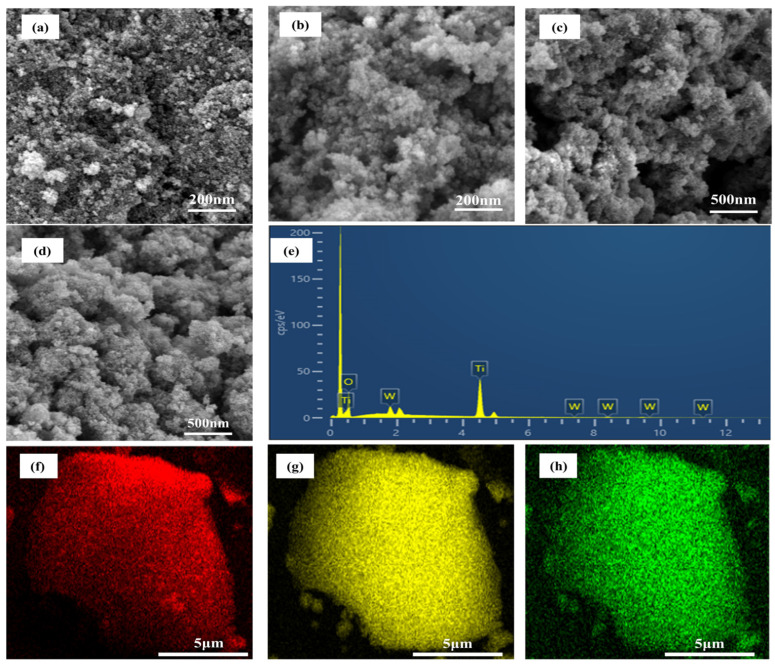
SEM images of pristine TiO_2_-Et (**a**), SEM images of W1-TiO_2_-Et (**b**), SEM images of W2-TiO_2_-Et (**c**), SEM images W3-TiO_2_-Et (**d**), EDS spectrum of W1-TiO_2_-Et (**e**), EDS mapping images of O (**f**), Ti (**g**), and W (**h**) on W1-TiO_2_-Et.

The SEM and EDS mapping images of the pristine TiO_2_ and tungsten-doped TiO_2_ are shown in Figure 3. It can be found that pristine TiO_2_ exhibits textural characteristics with a dominant presence of irregularly shaped aggregates accompanied by interparticle voids [44]. This observation highlights the agglomeration tendency of pristine TiO_2_ [20]. Tungsten-doped TiO_2_ with different tungsten precursors of WCl_6_, Na_2_WO_4_●2H_2_O, and AMT have been displayed in Figure 3b–d. Compared with Figure 3a, the tungsten-doped TiO_2_ samples were looser, which suppressed the tendency of agglomeration to some extent. Some studies suggest that this could prove advantageous for both adsorption and photocatalysis processes [45,46]. Compared with Figure 3b–d, W3-TiO_2_-Et exhibits a loosely structured morphology characterized by enhanced porosity, which potentially contributes to a significantly larger specific surface area compared to other tungsten-doped TiO_2_. The microscopic chemical ingredient analysis of the tungsten-doped TiO_2_ materials has been shown in Figure 3e. The patterns show the presence of titanium (Ti), oxygen (O), and tungsten (W) without any other element. The elements of Ti, O, and W are identified, confirming the presence of measured atomic percentage of 26.99, 71.61, and 1.40%, respectively. It can be clearly seen that the atomic percentage of oxygen is 2.7 times that of titanium, which may be due to the presence of oxygen functionalities remaining on the surface of the tungsten-doped TiO_2_ [35]. Figure 3f–h shows the elemental mapping of oxygen (O), titanium (Ti), and tungsten (W) in different colors on W1-TiO_2_-Et. The homogeneous distribution of three elements within the W1-TiO_2_-Et inferred the successful synthesis of tungsten-doped TiO_2_. This homogeneity suggests a well-integrated structure on materials which is crucial for the photocatalysis [29].

**Figure 4:**
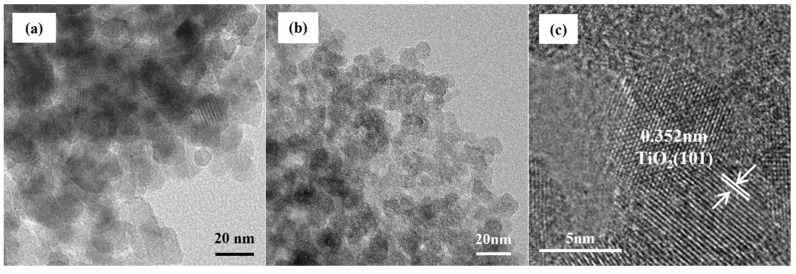
TEM images of TiO_2_-Et (**a**) and W1-TiO_2_-ET (**b**), HRTEM images of W1-TiO_2_-Et (**c**).

TEM and HRTEM techniques were used to analyse the morphology and microstructure of TiO_2_-ET and W1-TiO_2_-Et nanoparticles. In the TEM analysis (Figure 4), it was observed that the morphology of tungsten-doped TiO_2_ and pristine TiO_2_ nanoparticles is similar. Both materials are characterized by the presence of spherical nanoparticles, which aggregate to form larger clusters. The particle size distribution is between 6~10 nm, which is consistent with the calculation results of Scherrer’s equation (Table 1). The HRTEM image in Figure 4c revealed a fringe spacing of approximately 0.352 nm, which is consistent with the crystal growth direction of the anatase TiO_2_ (1 0 1) plane, as evidenced by XRD measurements of the sample.

As shown in Figures S2 and S3, the N_2_ adsorption and desorption isotherm curves were recorded to study the specific surface area and corresponding pore size distribution of the tungsten doped samples [47]. The IUPAC classification of the nitrogen adsorption and desorption isotherms for the examined samples revealed a type V pattern, indicating the mesoporous properties of the prepared material. Meanwhile, the pore size distribution of all the samples was mainly distributed in 0~50 nm, which also indicated the mesoporous properties of the prepared materials. As shown in Table 1, except for the tungsten source with Na_2_WO_4_●2H_2_O as the precursor, doping with other tungsten precursors increased the specific surface area, pore size, and pore volume of the materials and provided more active sites. The material with Na_2_WO_4_●2H_2_O as the precursor had little effect on the specific surface area, but the pore size of the material with Na_2_WO_4_●2H_2_O as the precursor was significantly increased compared to the pristine TiO_2_, which may improve the adsorption and photocatalytic ability of the materials. In Table 1, it was found that W3-TiO_2_-DMF had the largest specific surface area of 350.39 m^2^/g, while the pore volume and pore size of the samples with ethanol as a solvent were larger than those of the samples with DMF as a solvent. 

The crystalline phase of as-prepared samples was analyzed by X-ray photoelectron spectroscopy (XPS). In the XPS spectrum of Ti 2p, two XPS signals appear with binding energies of 458.8 and 464.5 eV, which are contributed by Ti 2p^1/2^ and Ti 2p^3/2^ and originate from the Ti^4+^ (Figure S4) [48,49,50]. Figure S4 shows that the binding energy at 529.88 eV corresponds to the crystal lattice oxygen of Ti–O or W–O, suggesting that W–O and Ti–O share O 1s orbitals in the W–O–Ti bond. The binding energy at 531.2 eV originates from hydroxyl groups bonded to Ti or W at the surface, and the binding energy near 532.0 eV originates from adsorbed water (H_2_O_ads_) or oxygen bonded to carbon (C–O) [29,51]. The O 1s binding energies of pristine TiO_2_ and tungsten-doped TiO_2_ materials were investigated in Figure S5, and it was found that the tungsten-doped TiO_2_ materials were shifted towards a higher binding energy in the O 1s orbital, which may be because the electronegativity of W (2.36) is greater than the electronegativity of Ti (1.54). The doping of TiO_2_ with tungsten can alter the electron cloud density of oxygen, leading to a slight shift to the binding energy towards to higher place.

**Figure 5:**
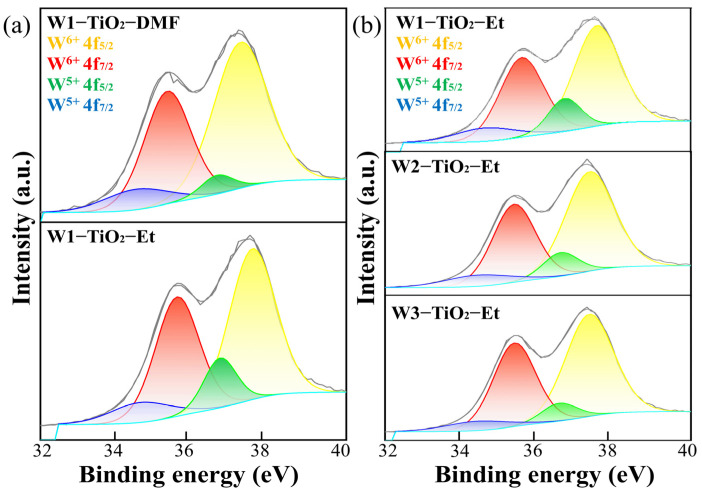
XPS spectra of W 4f region for (**a**) W1-TiO_2_-DMF and W1-TiO_2_-Et, (**b**) W1-TiO_2_-Et, W2-TiO_2_-Et, and W3-TiO_2_-Et.

As illustrated in Figure 5, the visual representation revealed that the samples synthesized using a range of tungsten precursors exhibited a co-doping pattern, characterized by the presence of both W^5+^ and W^6+^ ions. However, a different ratio of W^5+^ to W^6+^ was discerned among these samples. The disparity in the W 4f region between W1-TiO_2_-Et (WCl_6_), W2-TiO_2_-Et (Na_2_WO_4_●2H_2_O), and W3-TiO_2_-Et (AMT) was employed as an illustrative example. The W 4f peak of W1-TiO_2_-Et (WCl_6_) was deconvoluted into four peaks, W^6+^ 4f^5/2^ at 37.55 eV, W^6+^ 4f^7/2^ at 35.58 eV, W^5+^ 4f^5/2^ at 36.70 eV, and W^5+^ 4f^7/2^ at 34.62 eV [52,53], while the XPS spectrum of pristine TiO_2_ is flat in this region. This is consistent with the expected characteristic signature of pristine TiO_2_. The analysis reveals that the signals of W2-TiO_2_-Et (Na_2_WO_4_●2H_2_O) and W3-TiO_2_-Et (AMT) can be explicitly decomposed into four distinct peaks, each exhibiting comparable positions within the examined region. The W^5+^/W^6+^ ratios of W1-TiO_2_-Et, W2-TiO_2_-Et, W3-TiO_2_-Et, W1-TiO_2_-DMF, W2-TiO_2_-DMF, and W3-TiO_2_-DMF were 1:4.6, 1:5.2, 1:7.0, 1:7.0, 1:7.8, and 1:8.9, respectively. The study’s outcomes demonstrate the substantial influence of diverse tungsten precursors on the co-doping process involving W^5+^ and W^6+^. The investigation of tungsten precursors is crucial for understanding the physicochemical properties and photocatalytic performance of tungsten-doped TiO_2_. However, the intricacies of the mechanism remain unexplored, necessitating comprehensive investigation for a clearer understanding.

### 2.2. Photocatalytic Degradation of TC and CIP

**Figure 6:**
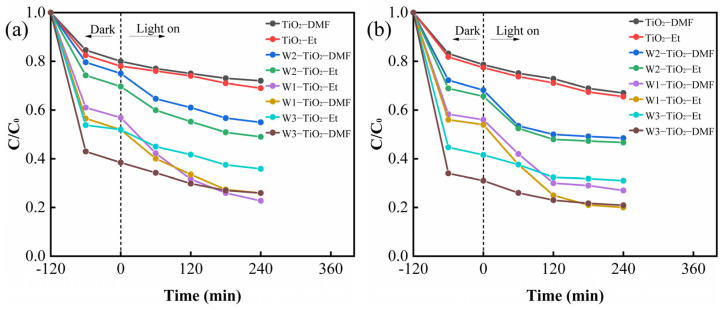
Removal curves of TC (50 mg/L) (**a**) and CIP (50 mg/L) (**b**).

The photocatalytic activity of the tungsten-doped TiO_2_ was evaluated by photodegradation of TC (50 mg/L) and CIP (50 mg/L). In contrast, the degradation of the TC and CIP solutions showed consistent results for the prepared samples. The activities of all W^5+^/W^6+^ co-doped samples were increased compared to the undoped TiO_2_. As shown in Figure 6a,b, the highest removal of TC and CIP was achieved by the prepared W1-TiO_2_-Et composites, and the removal rate of W1-TiO_2_-Et (77.24%, 80%) was more than 2.5 times that of pure TiO_2_ (31%, 32%). Meanwhile, in the dark, it was found that different tungsten precursors had a great influence on the adsorption effect, and high adsorption of TC and CIP by W3-TiO_2_-DMF was observed.

**Figure 7:**
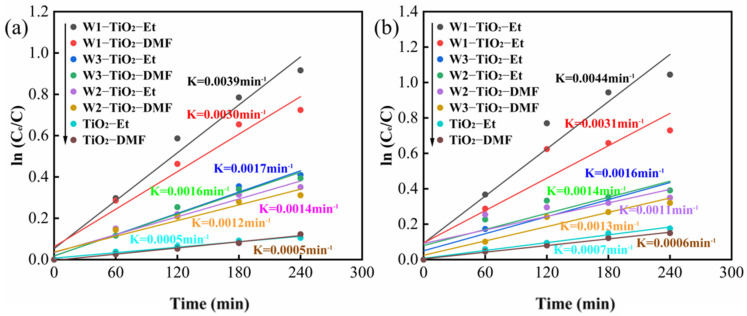
Apparent first order rate constant k (min^−1^) for TC (50 mg/L) (**a**) and CIP (50 mg/L) (**b**) photocatalytic degradation over the as-prepared samples.

The kinetic analyses of TC and CIP degradation were based on the fitting of pseudo-first order equations with kinetic constants as shown in Figure 7. During the photocatalytic degradation of TC and CIP, the highest K values of 0.0039 min^−1^ (Table 2) and 0.0044 min^−1^ (Table 3) were obtained for W1-TiO_2_-Et, respectively. The increase in K value after the addition of tungsten precursors indicates that the co-doping of W^6+^ and W^5+^ accelerated the degradation of TC and CIP in the composites. The co-doping promotes carrier migration and reduces the photogenerated electron-hole complexation efficiency [31].

In addition, we made comparisons with previous work. As shown in Table 4 and Table 5, a variety of photocatalysts for TC and CIP degradation are listed. By comparison, it was found that the larger concentration of TC and CIP was selected as the degradants in this work, and the prepared W^5+^/W^6+^ co-doped TiO_2_ composites showed high removal rates for both degradants.

**Figure 8:**
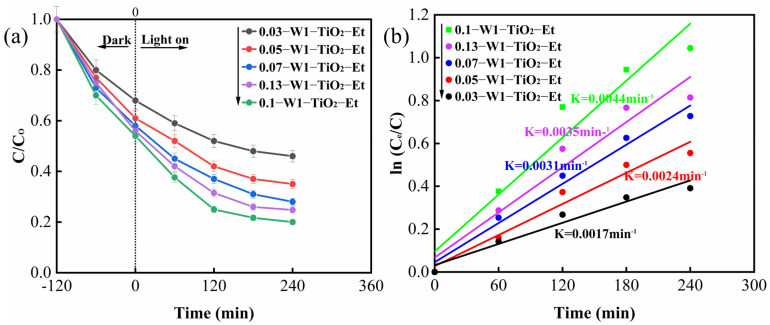
CIP removals curves under different W doping content (**a**), apparent first order rate constant k (min^−1^) for CIP photocatalytic degradation (**b**).

The effect of different W contents on the photocatalytic properties of tungsten-doped TiO_2_ composites was investigated as shown in Figure 8. Figure 8a shows the removal curves of CIP, and Figure 8b shows the apparent first-order rate constant k (min^−1^) for CIP. Figure 8a shows that the highest CIP removal rate (80%) of tungsten-doped TiO_2_ composites was achieved when Ti/W = 3:0.1, but when the W content was further increased, the removal rate decreased instead. Figure 8b also shows that the composites have the maximum K value for Ti/W = 3:0.1, indicating that Ti/W = 3:0.1 is the optimum W loading. The photocatalytic activity of tungsten-doped TiO₂ composites was observed to increase when the optimum tungsten loading was reached, beyond which certain surface reaction sites of photocatalytic activity may be hindered, thus limiting the reaction rate [29].

In summary, the photocatalyst with ammonium metatungstate (AMT) as the tungsten precursor and DMF as the solvent exhibited the strongest adsorption capacity for ciprofloxacin (CIP) and tetracycline (TC) in the dark (52% and 48% in 2 h). However, the photocatalyst with WCl_6_ as the tungsten precursor and ethanol as the solvent (W1-TiO_2_-Et) showed the highest photodegradation performance for ciprofloxacin (CIP) and tetracycline (TC). The photodegradation performance to tetracycline (TC) on W1-TiO_2_-Et was 2.3, 2.8, and 7.8 times that of AMT, Na_2_WO_4_●2H_2_O as the tungsten precursors and pristine TiO_2_, respectively. The optimum tungsten loading was also investigated in this work, and it was found that Ti/W = 3:0.1 is the optimum tungsten loading.

### 2.3. Mechanism

It is worth highlighting some facts about tungsten-doped TiO_2_ which exhibited higher photocatalytic efficiency for the degraded TC and CIP, though the mechanism is far from understood.

**Figure 9:**
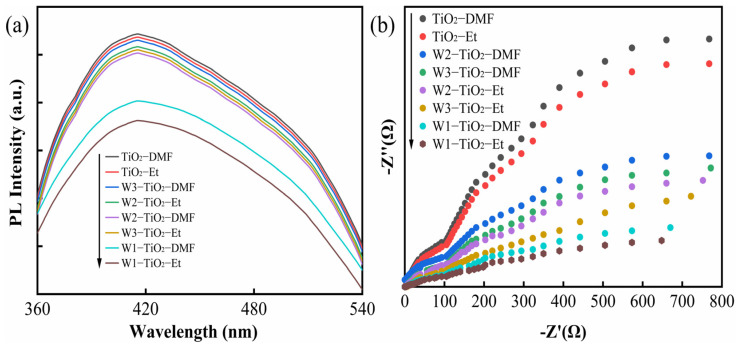
PL spectra of as-prepared samples (**a**), EIS Nyquist plots of pure TiO_2_ and W-TiO_2_ composites (**b**).

As illustrated in Figure 7, the photocatalytic efficiencies of the different tungsten-doped TiO_2_ samples are significantly different. XPS analysis (Figure 5) revealed that these materials exhibited different ratios of W^5+^ and W^6+^. The photocatalytic efficiency of the prepared samples may be related to the ratio of W^5+^ and W^6+^, and co-doping promotes carrier migration and reduces the photogenerated electron-hole complex efficiency. To confirm this hypothesis, electrochemical impedance spectroscopy (EIS) and photoluminescence (PL) analyses have been performed, and the results are shown in Figure 9a,b.

The findings indicated that tungsten-doped TiO_2_ exhibited a diminished photogenerated carrier radius and an extended photogenerated carrier lifetime in comparison to pristine TiO_2_. This suggests that the separation rate and migration of photogenerated carriers in the W^5+^/W^6+^ co-doped composites are more rapid than in pristine TiO_2_. W1-TiO_2_-Et has the smallest photogenerated carrier radius and the longest photogenerated carrier lifetime, which confirms that the W1-TiO_2_-Et has the highest photocatalytic activity. Meanwhile, it can be seen from Figure 5 that the W^5+^/W^6+^ ratio of W1-TiO_2_-Et is the largest; the higher percentage of W^5+^ indicates the higher oxygen vacancy concentration [53], which accelerates the migration rate of photogenerated carriers. The higher concentration of oxygen vacancies also makes it easier to adsorb water to form surface hydroxyls, which promotes the photocatalytic efficiency [53]. It is believed that the phenomenon should be influenced by the molecular configuration of the tungsten precursors. Tungsten in AMT and Na_2_WO_4_●2H_2_O is surrounded by oxygen and hydroxyl groups/H_2_O, respectively. In contrast, tungsten in tungsten (VI) chloride (WCl_6_) is also in the +6 oxidation state but is coordinated by chlorine atoms. The difference in coordination environment between these tungsten compounds results in the diverse ways in which tungsten can be bonded in the synthesis of tungsten-doped TiO_2_, influenced by the steric hindrance effect and surface hydroxyl group. The combined effects underscore the efficiency of photocatalytic degradation in the removal of TC and CIP.

The solvent also impacted the photocatalytic efficiency of the synthesized samples obviously. Figure 7 shows that tungsten-doped TiO_2_ synthesized with ethanol exhibited enhanced photocatalytic efficiency compared to those synthesized in DMF. This phenomenon may be attributed to the varying conformations exhibited by ethanol and DMF molecules. As mentioned earlier, compared to DMF, using ethanol as solvent can increase the crystallinity, pore size, and pore volume of the tungsten-doped TiO_2_ materials. This facilitates charge transfer from the center to the surface and increases the active sites, thus improving the photocatalytic activity. In addition, the gel formation process was faster in the DMF solvent than in the ethanol solvent during the preparation process, which may be due to the larger polarity and dielectric constant of DMF compared to ethanol. The reaction was slower when ethanol was used as a solvent and the nanoparticles nucleated uniformly in ethanol solvent [60]. Ethanol can be used as a very dispersive solvent by the reaction and ethanol as a solvent plays an important role in controlling the crystal growth [60].

**Figure 10:**
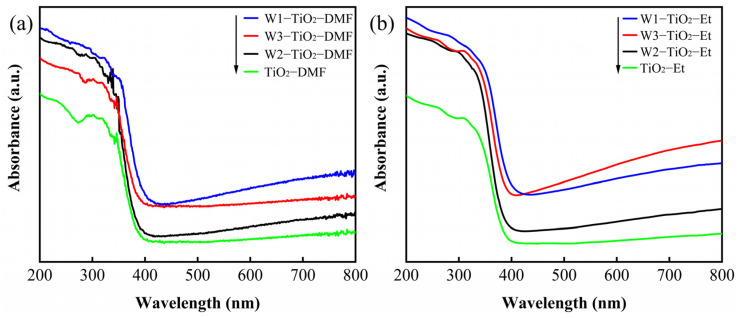
UV-vis diffuse reflectance spectra, (**a**) W1-TiO_2_-DMF, W2-TiO_2_-DMF and W3-TiO_2_-DMF, (**b**) W1-TiO_2_-Et, W2-TiO_2_-Et and W3-TiO_2_-Et.

Figure 10 shows the UV-vis absorption spectra of tungsten-doped TiO_2_ with different tungsten precursors and solvents. In general, the absorption of tungsten-doped TiO_2_ was stronger than that of undoped TiO_2_ in visible light region. The red shift was observed with the incorporation of tungsten into TiO_2_ materials. The band gaps of the prepared tungsten-doped TiO_2_ are estimated by the Tauc plot as shown in Figure S7. Pristine TiO_2_ exhibited the E_g_ values about 3.3 eV, which is consistent with the value reported in literature [61]. It is obvious that doping TiO_2_ with tungsten slightly modifies the TiO_2_ absorption edge in the visible region. This observed phenomenon has been elucidated by the quantum confinement effect [34,62]. It is true that tungsten doping in TiO_2_ has been observed to reduce the band gap of photocatalysts, which is attributed to create new energy levels within the band gap [49]. However, smaller particle size often leads to higher E_g_ values [13]. As the size of the particles decreases, the degree of quantization of energy levels is stronger, leading to an increased band gap [45]. That is, the narrow band gap and red-shifted adsorption edges of the samples analysed by UV-visible diffuse reflectance spectroscopy may indicate a better photocatalytic activity of the tungsten-doped TiO_2_ samples [29].

For the purpose of analysing the adsorption mechanism of the tungsten-doped TiO_2_ samples in the dark, the zeta potential of the tungsten-doped TiO_2_ samples have been measured (Figure S8), focusing on their surface charge characteristics.

The pka1, pka2, and pka3 of TC are 3.3, 7.7, and 9.7, respectively [63]. Positive charges dominate when the pH is below 3.3, both positive and negative charges are present when the pH is below 7.7 but above 3.3, and negative charges dominate when the pH is above 7.7 [60]. CIP has two pKa of 5.9 and 8.9 at pH values between 5.9 and 8.9 [64], at pH values below 5.9 the cation of CIP is dominant, and at pH values above 8.9, the anion of CIP is dominant [65,66]. This work is based on 50 ppm TC and 50 ppm CIP solutions at pH 3.2 and 5.3, so positive charges dominate in both 50 ppm TC and 50 ppm CIP solutions.

As shown in Table 6, the samples with AMT as the tungsten precursors had less positive surface charge in TC and CIP solutions (pH = 3.2, 5.3). According to the principle of anisotropic attraction and anisotropic repulsion, the sample with AMT as the tungsten precursor has a better adsorption effect on ciprofloxacin solution in the dark. From Table 1, it can be seen that the specific surface area of W3-TiO_2_-DMF is larger than the other samples; therefore, combined with the BET potential and zeta potential, it can be concluded that W3-TiO_2_-DMF has a stronger adsorption effect on the 50 ppm TC and 50 ppm CIP solutions, which is also consistent with the results in Figure 6.

**Figure 11:**
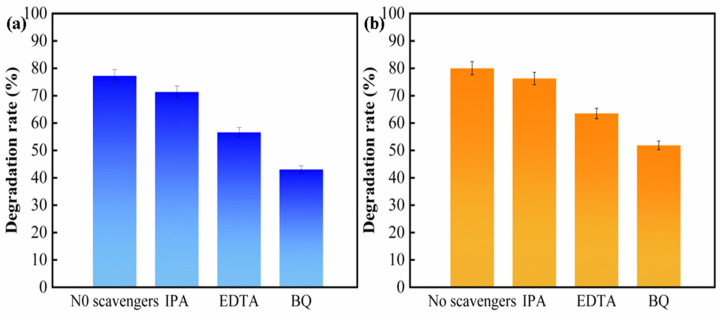
Free radical capture experiment TC (**a**), CIP (**b**).

Free radical trapping tests were carried out on W1-TiO_2_-Et as shown in Figure 11a,b to determine which are the active species in the photocatalytic process of W1-TiO_2_-Et [67]. The photodegradation efficiency decreased when IPA, EDTA-2Na, and BQ were added to the TC and CIP solutions. IPA (71.3%, 76.24%), EDTA-2Na (56.6%, 63.49), and BQ (43%, 51.86). This result indicates that ·O^2−^ and h^+^ are the main active species of W1-TiO_2_-Et photocatalysts during the photodegradation of CIP and TC [68].

**Figure 12:**
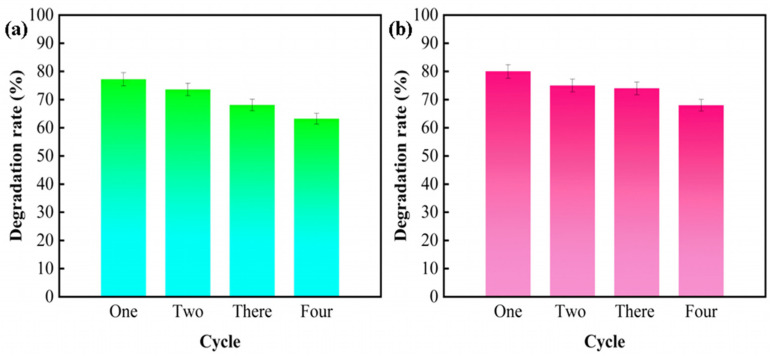
Recycling tests of degradation TC (**a**) and CIP (**b**) using W1-TiO_2_-Et.

To assess the reusability of the photocatalysts, the W1-TiO_2_-Et photocatalysts were subjected to four cycles of reusability tests. The results are shown in Figure 12: the photocatalyst exhibits excellent photocatalytic efficacy for TC and CIP degradation in all reusability tests. This proves that the sample is an excellent photocatalyst for reusability.

## 3. Materials and Methods

### 3.1. Chemicals and Reagents

Sodium tungstate dihydrate (Na_2_WO_4_●2H_2_O, ≥98%) and titanium tetraisopropoxide (TTIP, ≥98%) were purchased from Adamas-beta (Shanghai, China). Tungsten (VI) chloride (WCl_6_, 99%), ammonium metatungstate hydrate (AMT, 99.5%), N,N-dimethylformamide (DMF, 99.5%), ethanol (Et, ≥99.7%), nitric acid (HNO_3_, 65–68%), p-benzoquinone (BQ, 99%), ciprofloxacin (CIP, 98%), and tetracycline hydrochloride (TC, 99%) were purchased from Merck (Shanghai, China). Thylenediaminetetraacetic acid disodium salt (EDTA-2Na, ≥99%) and isopropanol (IPA, 99.7%) were purchased from Tianjin ZhiYuan Reagent Co., Ltd. (Tianjin, China). All chemicals were used without further purification.

### 3.2. Catalyst Preparation

In a typical procedure, 0.44 g of WCl_6_ and 3 drops of nitric acid were added to 38.6 mL of DMF/ethanol and stirred until the WCl_6_ was completely dissolved, and then 10 mL of TTIP and 2.5 mL deionized water were added slowly drop by drop to hydrolyze the TiO_2_ completely. The sealed sol-gel was aged at room temperature for 48 h, then calcined in a high temperature and pressure reactor at 265 °C for 2 h. Then, it was washed with deionized water and ethanol, dried, and collected. Composites featuring sodium tungstate (Na_2_WO_4_) and AMT as the tungsten precursors were synthesized using identical methodologies. Employing WCl_6_ as the tungsten precursor, the composite’s synthesis involved a systematic investigation using two kinds of solvents, W1-TiO_2_-DMF and W1-TiO_2_-Et. Similarly, for sodium tungstate (Na_2_WO_4_), the samples were denoted W2-TiO_2_-DMF and W2-TiO_2_-Et, and for AMT, W3-TiO_2_-DMF and W3-TiO_2_-Et, respectively. All tungsten-doped TiO_2_ materials referenced above exhibit a Ti: W molar ratio of 3:0.1. The sample synthesis flowchart is shown in Figure S1.

In order to study the effect of tungsten doping, other samples with different Ti: W = 0.03, 0.05, 0.07, 0.1, 0.13 composites were synthesized by the similar method using WCl_6_ as the tungsten precursors and ethanol as the solvent, and the samples were named as 0.03-W1-TiO_2_-Et, 0.05-W1-TiO_2_-Et, 0.07-W1-TiO_2_-Et, 0.1-W1-TiO_2_-Et, and 0.13-W1-TiO_2_-Et. For comparison, two TiO_2_ materials using ethanol and DMF as solvents which denoted as TiO_2_-Et and TiO_2_-DMF were prepared by similar method.

## 4. Conclusions

The successful synthesis of tungsten-doped TiO_2_ samples with tunable W^5+^/W^6+^ ratio using a simple sol-gel method. To explore the impact of tungsten precursors and solvents in the synthesis of tungsten-doped TiO_2_ on the photocatalytic degradation of selected pharmaceuticals and personal care products (PPCPs), tetracycline (TC), and ciprofloxacin (CIP) were chosen as targets. The increase in photocatalytic efficiency may be due to the synergistic effect of crystallinity, surface properties, molecular configuration of the solvent, and W^5+^/W^6+^ ratio of the samples, in which the co-doping of W^5+^/W^6+^ may play a more important role. The co-doping of W^5+^/W^6+^ accelerated the photogenerated carrier migration rate and increased the photogenerated carrier lifetime, resulting in a higher photocatalytic efficiency of the tungsten-doped TiO_2_ samples than that of the pristine TiO_2_. In addition, the distinct molecular configurations of ethanol and DMF as solvents during the synthesis process resulted in variations in the gel formation rate, polarity, and dielectric constant of the tungsten-doped TiO_2_. These differences ultimately affect the photocatalytic degradation of TC and CIP on the tungsten-doped TiO_2_. The radical trapping assay revealed that ·O^2−^ and h^+^ were found to be the main reactive species in the degradation process of TC and CIP. Repeated experiments showed that W1-TiO_2_-Et was a stable catalyst. In summary, the TC and CIP removal efficiency of tungsten-doped TiO_2_ suggest that it may be a promising candidate for the treatment of PPCPs under simulated sunlight. 

## Figures and Tables

**Table 1:** Physicochemical properties of tungsten-doped TiO_2_ samples.

Samples	Average Crystalline Size (nm)	S_BET_ (m^2^/g)	Band Gap Energy (eV)	Pore Volume (cm^3^/g)	Pore Size (A)
TiO_2_-DMF	10.2	145.12	3.32	0.29	7.18
TiO_2_-Et	10.8	143.23	3.32	0.33	10.45
W1-TiO_2_-DMF	8.2	205.60	3.13	0.22	34.01
W1-TiO_2_-Et	9.6	202.25	3.17	0.44	64.94
W2-TiO_2_-DMF	9.9	148.63	3.26	0.29	58.01
W2-TiO_2_-Et	10.1	147.98	3.29	0.37	70.69
W3-TiO_2_-DMF	6.4	350.39	3.24	0.37	37.08
W3-TiO_2_-Et	7.9	200.82	3.21	0.54	58.21

**Table 2:** The corresponding kinetic constant of TC degradation (except for adsorption).

Samples	TiO_2_-DMF	TiO_2_-Et	W1-TiO_2_-DMF	W1-TiO_2_-Et	W2-TiO_2_-DMF	W2-TiO_2_-Et	W3-TiO_2_-DMF	W3-TiO_2_-Et
K (min^−1^)	0.0001	0.0002	0.0030	0.0039	0.0012	0.0014	0.0016	0.0017

**Table 3:** The corresponding kinetic constant of CIP degradation (except for adsorption).

Samples	TiO_2_-DMF	TiO_2_-Et	W1-TiO_2_-DMF	W1-TiO_2_-Et	W2-TiO_2_-DMF	W2-TiO_2_-Et	W3-TiO_2_-DMF	W3-TiO_2_-Et
K (min^−1^)	0.0001	0.0002	0.0031	0.0044	0.0011	0.0014	0.0013	0.0016

**Table 4:** Lists a variety of photocatalysts for TC degradation.

Sample	Dosage (g/L)	Concentration (mg/L)	Time (min)	Removal Rate (%)	Year	Author	Reference
Bi/BiVO_4_	0.5	10	60	74.7	2019	Nianjun Kang	[54]
2D/2D BiOCl/g-C_3_N_4_	0.1	10	30	97.1	2020	Yuwei Sun	[55]
Bi_2_O_3_/Ti^3+^—TiO_2_	0.2	10	200	96.5	2020	Tao Tang	[56]
This work	0.4	50	360	77.2	2024	----	----

**Table 5:** Lists a variety of photocatalysts for CIP degradation.

Sample	Dosage (g/L)	Concentration (mg/L)	Time (min)	Removal Rate (%)	Year	Author	Reference
Ti^3+^/N-TiO_2_	0.43	0.5	70	100	2020	Mansour Sarafraz	[57]
Carbon-doped TiO_2_	1	50	360	35	2015	Jian-Wen Shi	[58]
Fe-N-TiO_2_	0.6	20	360	70	2020	Totsaporn Suwannaruang	[59]
This work	0.4	50	360	80	2024	----	----

**Table 6:** Zeta potential of as- prepared samples.

pH	W1-TiO_2_-DMF (mV)	W1-TiO_2_-Et (mV)	W2-TiO_2_-DMF (mV)	W2-TiO_2_-Et (mV)	W3-TiO_2_-DMF (mV)	W3-TiO_2_-Et (mV)
3	21.6	22.1	5.81	8.5	4.67	3.86
5	0.668	−2.13	−12.4	−10.6	−12.2	−13.1
7	−18.3	−19.3	−18.2	−18.8	−19.2	−20.1
9	−23.7	−23.4	−22.1	−23.1	−23.3	−24.2
11	−29.9	−30	−28.7	−30	−31.6	−32.4

## Data Availability

The experimental data used to support the results of this study are available in the article and in the Supplementary Materials.

## Supplementary Materials

The following supporting information can be downloaded at: https://www.mdpi.com/article/10.3390/molecules29174164/s1, S1: Instruments and equipment; S2: Measurement of photocatalytic activity; Figure S1: Sample synthesis flowchart; Figure S2: Surface area analysis of W-TiO_2_ composites. N_2_ adsorption/desorption isotherms of (a) TiO_2_-DMF, (b) W1-TiO_2_-DMF, (c) W2-TiO_2_-DMF, (d) W3-TiO_2_-DMF; Figure S3: Surface area analysis of W-TiO_2_ composites. N_2_ adsorption/desorption isotherms of (a) TiO_2_-Et, (b) W1-TiO_2_-Et, (c) W2-TiO_2_-Et, (d) W3-TiO_2_-Et; Figure S4: XPS spectra of O 1S and Ti 2p region for W1-TiO_2_-Et; Figure S5: XPS spectra of O 1S region for W1-TiO_2_-DMF, W1-TiO_2_-Et, TiO_2_-Et, W3-TiO_2_-Et and W2-TiO_2_-Et; Figure S6: Adsorption/desorption curves in the dark (a) Tc, (b) CIP; Figure S7: plots of (ahv)2−hυ (a–d); Figure S8: Zeta potential of as-prepared samples (a,b).
