# Supplementary material for: Heterogeneous Photocatalytic Degradation of Selected Pharmaceuticals and Personal Care Products (PPCPs) Using Tungsten Doped TiO2: Effect of the Tungsten Precursors and Solvents
Source: Molecules. 2024 Sep 3;29(17):4164. doi: 10.3390/molecules29174164 (PMC11397017; doi:10.3390/molecules29174164)
Supplement: Supplementary file 1 [file molecules-29-04164-s001.zip › molecules-3125890-supplementary.pdf]

Supplementary Materials

Heterogeneous Photocatalytic Degradation of Selected Pharmaceuticals and Personal Care Products (PPCPs) Using Tungsten Doped TiO2: Effect of the Tungsten Precursors and Solvents

Kunyang Li, Jing Li, Fengying Luo, Yuhua Yu, Yepeng Yang and Yizhou Li *

Yunnan Key Laboratory of Metal-Organic Molecular Materials and Device, School of Chemistry and Chemical Engineering, Kunming University, Kunming 650214, China

* Correspondence: zh111111ou@kmu.edu.cn

S1 Instruments and equipment

The crystalline phase analysis of the prepared tungsten-doped TiO_2 composites was carried out by X-ray powder diffraction (XRD, D2 PHASER, Germany). The specific surface area (BET) and pore structure of the material were measured on a surface area and porosity analyzer (Micromeritics, ASAP 2460, USA). X-ray photoelectron spectroscopy (XPS, Thermo Scientific K-Alpha+, USA) was employed to analyze the chemical state of elements within the samples. The ultraviolet-visible diffuse reflectance spectra and absorbance of the reaction solutions and powdered materials were assessed with a UV-2450 photometer (Shimadzu, Kyoto, Japan). Zeta potential was quantified with a nanoparticle size and zeta potential analyzer (Malvern Zetasizer, Nano ZS90, UK). Electrochemical impedance spectroscopy (EIS) data were acquired for the materials with the CS2350H electrochemical workstation (CORRTEST, Wuhan, China). Transmission electron microscopy (TEM) and high-resolution TEM (HRTEM) analyses were performed on the JEM-F200 instrument (JEOL, Japan) to examine the internal morphology and structure of the materials.

Scanning electron microscopy (SEM) and X-ray energy dispersive spectroscopy (EDS) mapping were utilized on the Sigma 300 instrument (ZEISS, Germany) to analyze the material's surface morphology and elemental distribution. Photogenerated carrier lifetimes were assessed through the analysis of photoluminescence (PL) spectra of the composites, employing the FS5 fluorescence spectrometer (Edinburgh Instruments Ltd, UK).

We used Jade software to calculate the crystallinity of the sample. Refer to Equation (1)

$$X_c = \frac{I_c}{I_c + I_a} \times 100\% \qquad (1)$$

X_c is the crystallinity of the test sample, I_c is the diffraction integral intensity of the crystalline part, and I_a is the diffraction integral intensity of the crystalline-free part.

In addition, Scherrer's formula was used to calculate the average crystalline size, which was calculated from equation (2).

$$D_{hkl} = k\lambda \div \beta Cos_{\theta} \qquad (2)$$

D_{hkl} is the average crystalline size, λ equals 0.15405981 nm, θ and β are the diffraction angle and the full width of the half-peak, k=0.89.

S2 Measurement of Photocatalytic activity

The adsorption and photocatalytic degradation performance of tungsten-doped TiO_2 composites were evaluated using TC and CIP as model PPCPs. A total of 20 mg of photocatalysts and 50 mL of PPCPs solution were mixed in a 50mL glass vessel with a flat quartz bottom. Prior to illumination, the mixture reached an equilibrium adsorption state via stirring in the dark for 120 minutes. After the mixture reached equilibrium adsorption (Figure S6), 2.0 ml of the reaction suspension was collected and filtered by a 0.22 μm membrane filter. The photocatalytic reaction was carried out under the xenon lamp (500 W, wavelength ranged of 170 to 700nm). During the experiment,

the water circulation pump should be turned on to keep the reactor at room temperature. In the presence of light, 2.0 ml of the reaction suspension was collected at 60-minute intervals, filtered through a 0.22-μm membrane filter, and irradiated continuously for 240 minutes. The removal efficiency of TC and CIP was calculated employing Equation (3), with C_0 denoting initial absorbance and C representing instantaneous absorbance at each time point. The first-order rate constant, k (min^{-1}), was calculated from Equation (4), where Ce referred to the equilibrium adsorption of TC and CIP. C is the instantaneous adsorption at light irradiation time t.

$$\text{Removal}(\%) = \frac{C_0 - C}{C} \tag{3}$$

$$\ln\left(\frac{C_e}{C}\right) = kt \tag{4}$$

The primary reactive oxidation species for the photodegradation process were identified by the scavengers. Isopropanol (IPA, 10ml), p-benzoquinone (BQ, 10 mM), and disodium ethylenediamine tetraacetate (EDTA-2Na, 10 mM) were used as scavengers for hydroxyl radicals (·OH), superoxide radicals ($\cdot O^{2-}$) species, and holes (h^+), respectively.

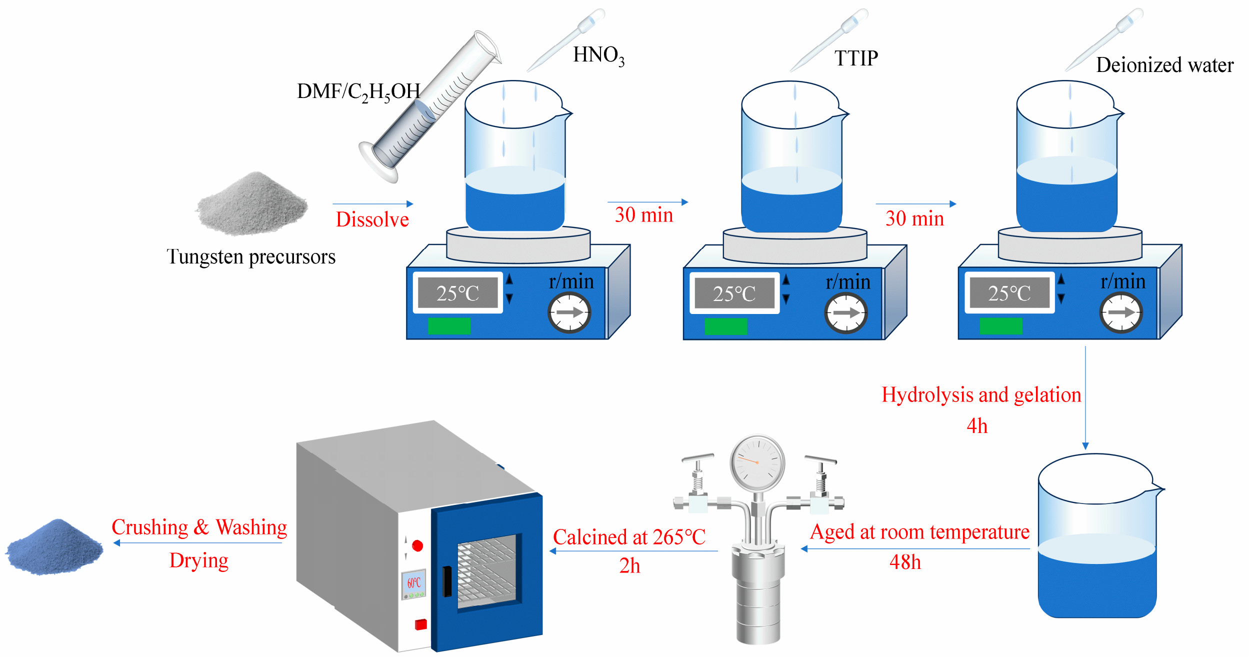

Figure S1. Sample synthesis flowchart

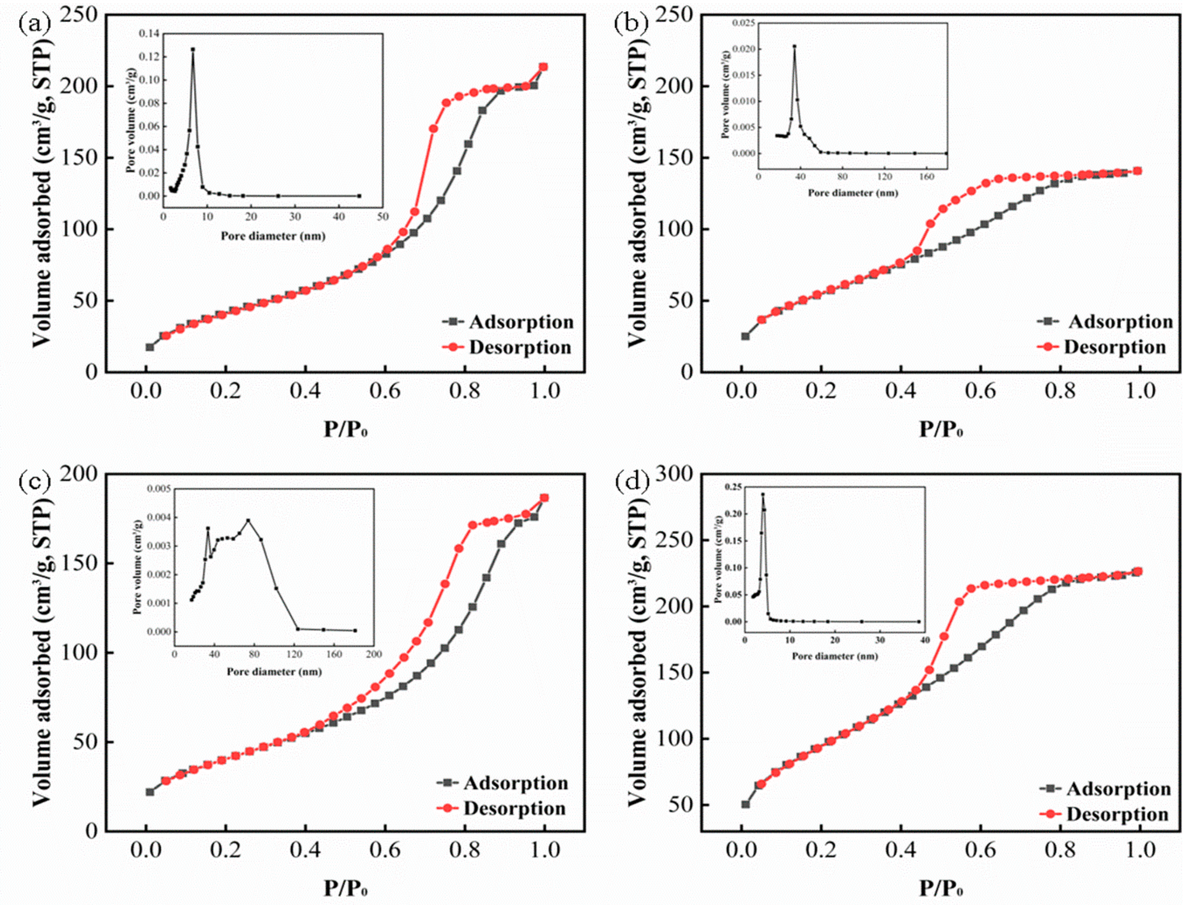

Figure S2. Surface area analysis of W−TiO_2 composites. N_2 adsorption/desorption isotherms of (a) TiO_2−DMF, (b) W1−TiO_2−DMF, (c) W2−TiO_2−DMF, (d) W3−TiO_2−DMF

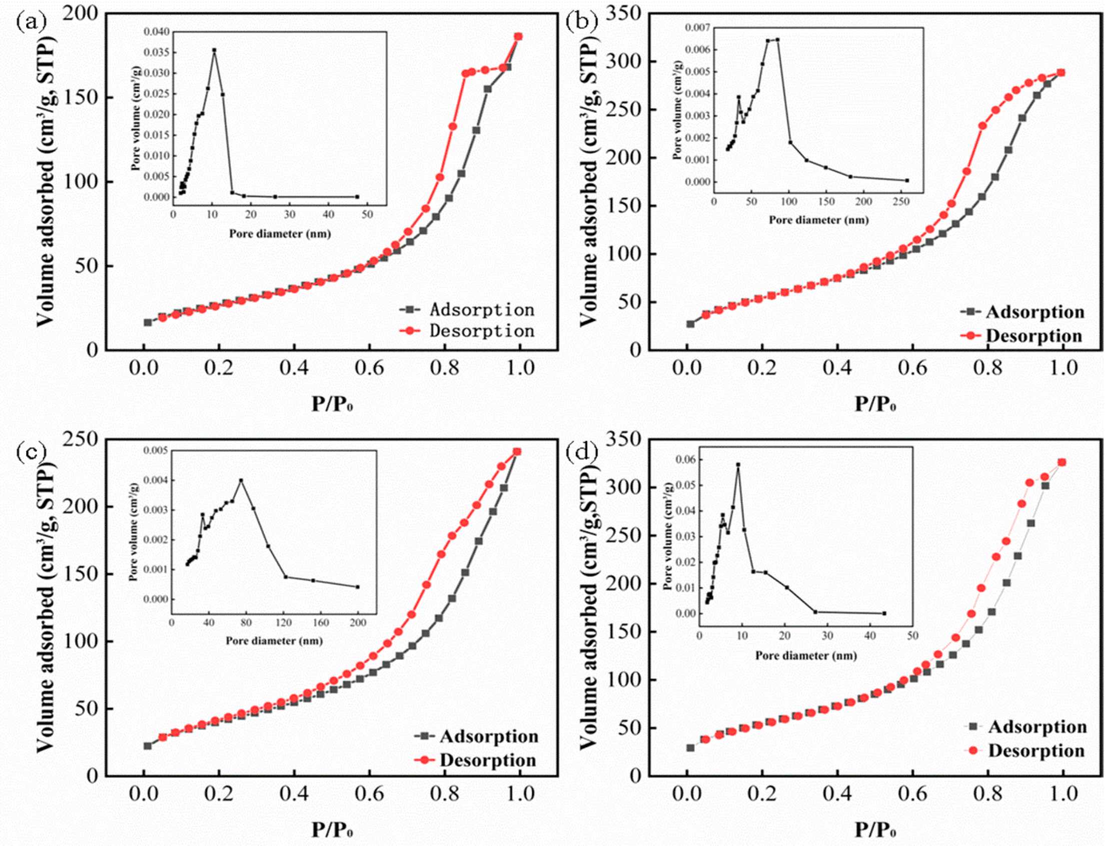

Figure S3. Surface area analysis of W−TiO_2 composites. N_2 adsorption/desorption isotherms of (a) TiO_2−Et, (b) W1−TiO_2−Et, (c) W2−TiO_2−Et, (d) W3−TiO_2−Et

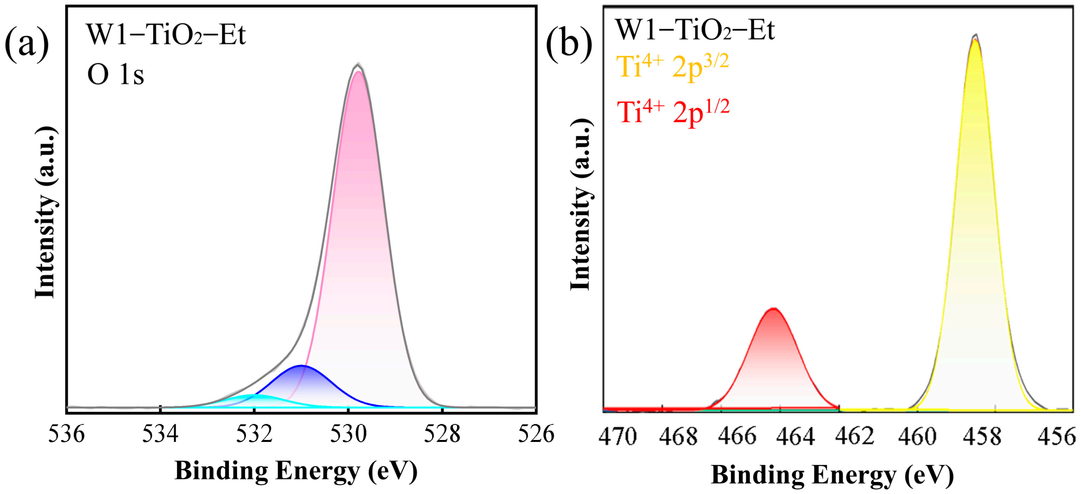

Figure S4. XPS spectra of O 1S and Ti 2p region for W1−TiO_2−Et

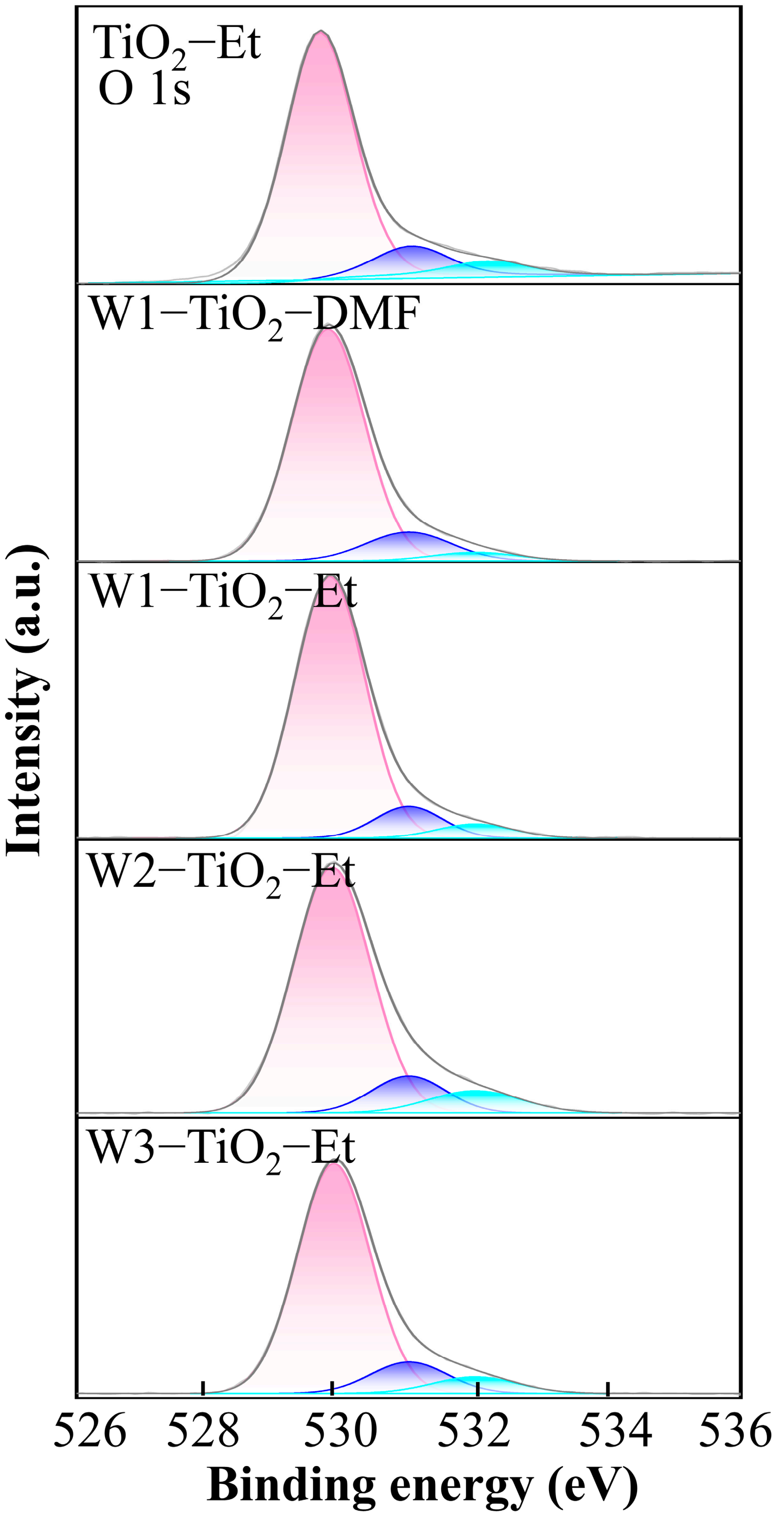

Figure S5. XPS spectra of O 1S region for W1−TiO_2−DMF, W1−TiO_2−Et, TiO_2−Et, W3−TiO_2−Et and W2−TiO_2−Et

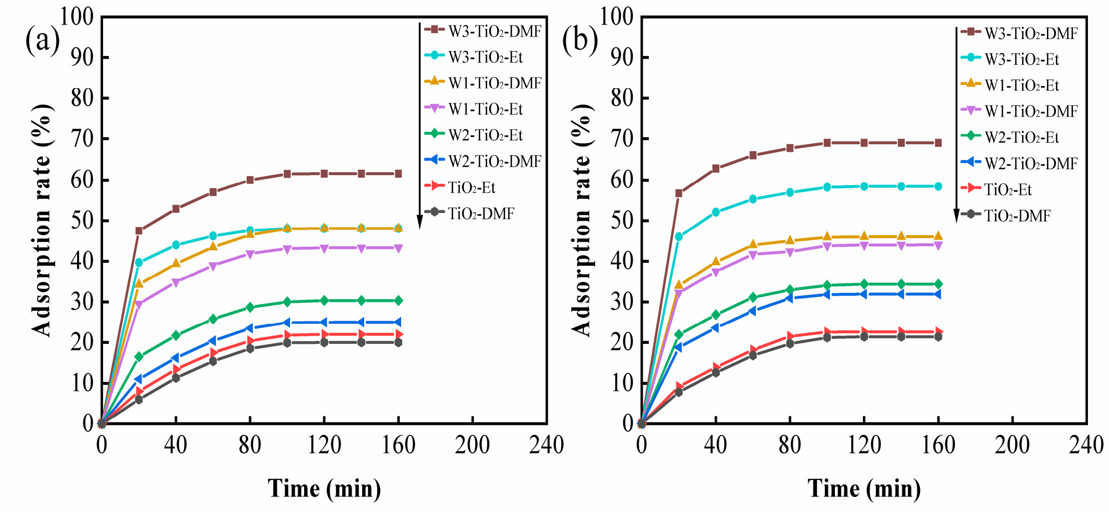

Figure S6. Adsorption/desorption curves in the dark (a) Tc, (b) CIP

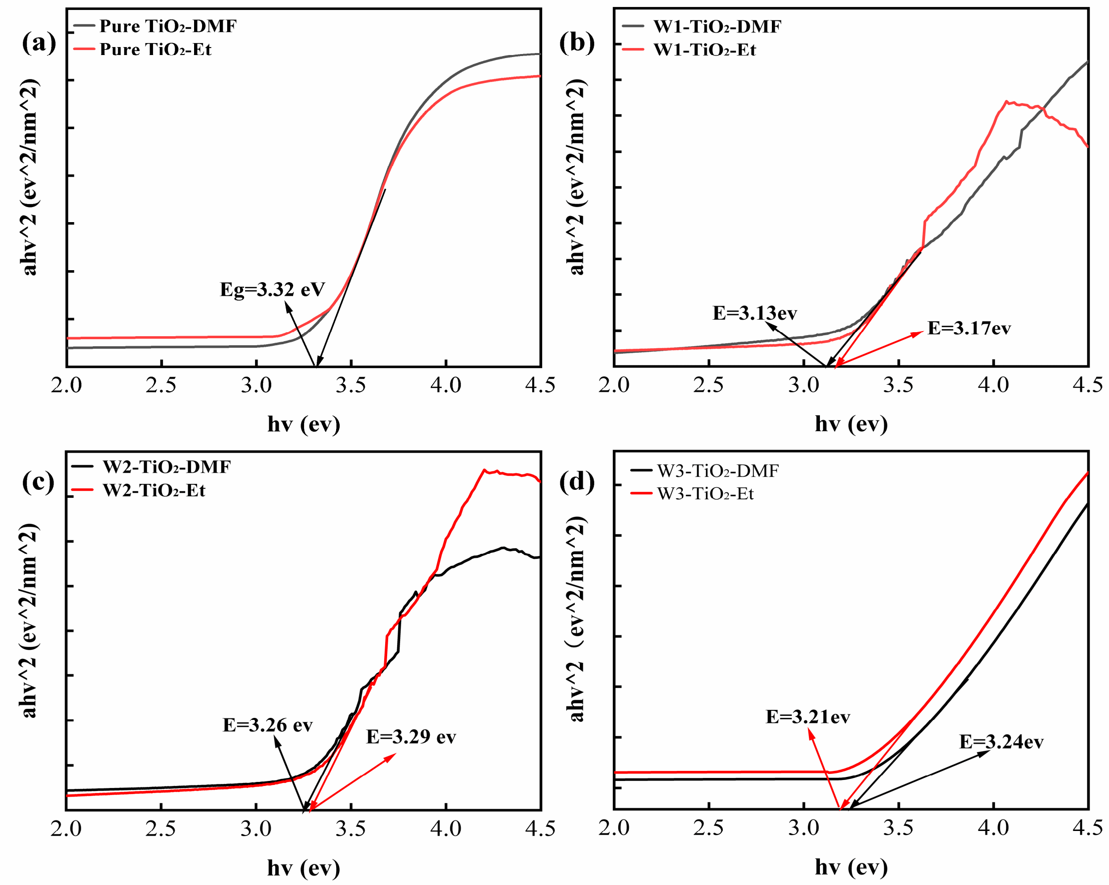

Figure S7. plots of $(ahv)^2$−hυ (a. b. c. d.)

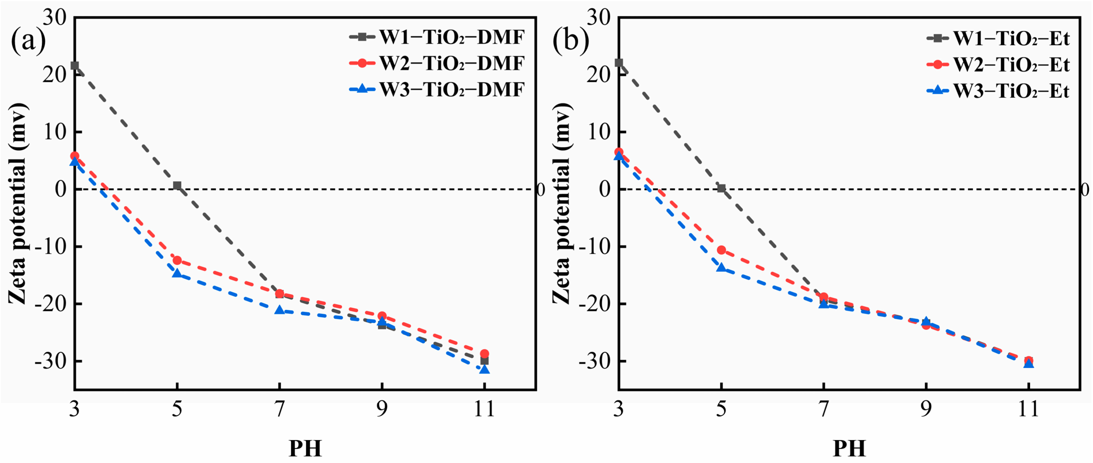

Figure S8. Zeta potential of as−prepared samples (a. b.)
